# Is there a difference between GBS triggered by COVID-19 and those of other origins?

**DOI:** 10.1186/s41983-022-00486-6

**Published:** 2022-05-15

**Authors:** Vanja Radišić, Mirjana Ždraljević, Stojan Perić, Branka Mladenović, Branislav Ralić, Dejana R. Jovanović, Ivana Berisavac

**Affiliations:** 1 Neurology Clinic, University Clinical Center of Serbia, Dr. Subotic Street 6, 11 000 Belgrade, Serbia; 2 Faculty of Medicine, University of Belgrade, Dr. Subotic Street 8, 11 000 Belgrade, Serbia; 3 Physical Medicine and Rehabilitation Clinic, University Clinical Center of Serbia, Pasterova Street 2, 11 000 Belgrade, Serbia; 4 Neurology Department, CHC Zvezdara, Presevska Street 31, 11 000 Belgrade, Serbia

**Keywords:** Guillain–Barré syndrome, COVID-19, Outcome, Functional disability

## Abstract

**Background:**

Since the outbreak of the coronavirus disease 2019 (COVID-19), an increasing number of Guillain–Barré syndrome (GBS) cases following the infection has been reported. The aim of our study was to detect patients with GBS treated in our hospital over a 1-year period and to compare the characteristics and outcomes of those triggered by COVID-19 with the rest of GBS patients. Our prospective study included 29 patients who were diagnosed with GBS from March 2020 to March 2021. Based on the preceding event, patients were stratified as post-COVID-19 and non-COVID-19. The GBS disability scale (GDS) was used to assess functional disability.

**Results:**

We identified 10 (34.5%) patients with post-COVID-19 GBS and 19 (65.5%) patients with non-COVID-19 GBS. The median time from the preceding event to the symptoms onset was longer in post-COVID-19 than in non-COVID-19 GBS patients (*p* = 0.04). However, the time from the symptom onset to the nadir did not differ (*p* = 0.12). GDS at admission, as well as at nadir, did not differ between these two groups. The level of proteinorrachia was higher in post-COVID-19 GBS patients (*p* = 0.035). The most frequent subtype of GBS in both groups was acute inflammatory demyelinating polyneuropathy (AIDP). GDS score at discharge (*p* = 0.56) did not differ between two study groups.

**Conclusions:**

There was no difference in clinical and electrophysiological features, disease course, and outcome in post-COVID-19 compared with non-COVID-19 GBS patients.

## Background

Coronavirus disease 2019 (COVID-19) is a systemic disorder caused by severe acute respiratory syndrome coronavirus 2 (SARS-CoV-2) [1]. After the first case of COVID-19 infection had been reported in Wuhan, China, in December 2019, SARS-CoV-2 has been spread rapidly, resulting in a global pandemic [2]. So far, numerous neurological manifestations associated with SARS-CoV-2 infection have been reported in the literature [3]. As COVID-19 is a systemic disorder, these manifestations may be due to involvement of the central nervous system, peripheral nervous system and/or muscles [4]. Some of these neurological manifestations, such as encephalitis, meningitis, acute cerebrovascular disease, and Guillain–Barré syndrome (GBS), are worrying due to the risk of long-term disability and high mortality [3]. Although some researches have been shown that SARS-CoV-2 has neuroinvasive abilities and that certain autoimmune diseases are associated with this infection, the exact mechanism of neurological manifestations occurring during or shortly after COVID-19 remains unclear [5–7].

GBS is an immune-mediated disease of the peripheral nerves and their roots, which typically presents with rapidly progressive bilateral limb weakness, sensory symptoms, and decreased or absent tendon reflexes [8]. It is a rare disease with an annual global incidence of approximately 0.81 to 1.89 per 100,000 person-years [9]. GBS is usually triggered by infection and therefore an increased incidence is expected during the outbreak of infectious diseases [8, 10]. Although an increasing number of GBS cases following COVID-19 have been reported worldwide, the link between GBS and COVID-19 remains controversial [11–13].

The aim of our study was to detect patients with GBS following COVID-19 in the cohort of GBS patients treated in our centre and to compare their clinical characteristics and outcomes with non-COVID-19 GBS patients.

## Methods

Our prospective study included 29 patients above the age of 18 diagnosed with GBS in our tertiary medical centre from March 2020, when the first case of COVID-19 was reported in Serbia [14], to March 2021. Forming of the GBS registry was approved by the Ethical Committee of the Faculty of Medicine (November 20th 2013, reference number: 29/XI-7), University of Belgrade and informed consent was signed by all patients.

Diagnosis of GBS was established using the Brighton Collaboration GBS Working Group criteria [15]. GBS was diagnosed with level 1 of diagnostic certainty in 12 patients and with level 2 in the remaining 17. The occurrence of the preceding event within 3–42 days before the onset of the first neurological symptoms was considered relevant [16]. Based on the known preceding event, patients were divided into two groups: GBS following COVID-19 (post-COVID-19 GBS) and non-COVID-19 GBS. Post-COVID-19 GBS group included patients who had positive history for COVID-19 confirmed via positive nasopharyngeal swab for viral RNA and positive serological test for specific SARS-CoV-2 IgG and/or IgM antibodies within 6 weeks before onset of neurological symptoms.

For each patient sociodemographic and clinical data, including gender, age, comorbidities, preceding event, cranial nerves involvement, presence of the limb weakness and sensory disturbances at the admission, course of the disease, presence of autonomic dysfunction during hospitalization, need for mechanical ventilation (MV) and its duration, diagnostic and laboratory findings, and therapy modalities were collected from the medical records. Muscle strength at admission was assessed by the Medical Research Council sum score (MRC-SS) [17]. GBS disability score (GDS) was used in order to determine functional disability at the admission, at nadir and on discharge [18]. Lumbar puncture was performed in order to determine cell count and protein level in the cerebrospinal fluid (CSF). Nerve conduction studies (NCS) were performed to assess the subtype of GBS (acute inflammatory demyelinating polyneuropathy—AIDP, acute motor axonal neuropathy—AMAN, acute motor and sensory axonal neuropathy—AMSAN) [19]. Nerve conduction studies were performed using an electromyoneurograph Medelec^®^ Synergy, Oxford Instruments, UK, manufactured in 2006.

Normality of data was tested by the Kolmogorov–Smirnov test. Continuous variables were presented using descriptive statistical modalities: mean ± standard deviation (SD) or median with interquartile range (IQR), while categorical variables were reported as absolute numbers and percentages. Statistical analyses were performed using Fisher’s exact test for categorical variables, and Mann–Whitney *U* test for continuous variables, as appropriate. Significance was defined as *p* < 0.05. Statistical analysis was performed using the IBM SPSS Statistics 17 (IBM, Armonk, New York, USA, 2017).

## Results

During a one-year period we identified 10 (34.5%) patients with post-COVID-19 GBS and 19 (65.5%) patients with non-COVID-19 GBS. Post-COVID-19 GBS patients had SARS-CoV2 infection confirmed by positive PCR of the nasopharyngeal swab and positive serum-specific IgG SARS-CoV-2 antibodies at the predefined time period. Our study did not include any patient with an active COVID-19 infection. Furthermore, negative nasopharyngeal swabs for SARS-CoV2, as well as pulmonary imaging (chest X-ray, pulmonary CT scan) without signs of interstitial pneumonia were required for all of our patients at admission. Regarding COVID-19 symptoms, 8 out 10 patients had symptoms of upper respiratory tract infection accompanied by fiver and received medical care at home, while 2 patients were admitted to specialized COVID-19 treating hospitals due to bilateral viral pneumonia. Five patients (50%) reported anosmia. All patients were treated according to Serbian National Protocol for COVID-19 infection and duration of treatment was determined by pulmonologist or infectologist. Patients who had pneumonia were treated with low doses of corticosteroids during hospitalization. Among patients with non-COVID-19 GBS, preceding factor was identified in 16 out of 19 patients (84.2%). The most common cause of GBS in this group was respiratory infection (8/19, 42.1%), whereas gastrointestinal infection was identified in 5 patients (26.3%). One patient has urinary tract infection, one has previous myocardial infarction and one had finger injury followed by surgical amputation.

Sociodemographic and clinical data of investigated post-COVID-19 GBS and non-COVID-19 GBS patients are shown in Table 1. Mean age of post-COVID-19 GBS patients was 55.2 ± 14.8 and of non-COVID-19 GBS patients 56.5 ± 15.7 (*p* = 0.84). Men accounted for 50% of patients in post-COVID-19 GBS group and 73.7% in non-COVID-19 GBS group (*p* = 0.24).The median time from the preceding event to the symptom onset was longer in post-COVID-19 than in non-COVID-19 GBS patients (21.5 days vs. 12 days, *p* = 0.04). There were no difference in MRC-SS (*p* = 0.17) and GDS (*p* = 0.7) at admission between these two groups. The time from the symptom onset to the nadir did not differ in post-COVID-19 and non-COVID-19 GBS patients (10 days vs. 8 days, *p* = 0.05) and there was no difference in the GDS at nadir between these two groups (*p* = 0.67).

**Table 1:** Demographic, clinical and diagnostic features of post-COVID-19 and non-COVID-19 GBS cases

Variables	Post-COVID GBS *n* = 10	Non-COVID GBS *n* = 19	*p*
Age, mean ± SD	55.2 ± 14.8	56.5 ± 15.7	0.836
Male gender, *n* (%)	5 (50%)	14 (73.7%)	0.244
Comorbidities, *n* (%)	7 (70%)	10 (52.6%)	0.449
Number of comorbidities, median (IQR)	1 (0–2)	1 (0–2)	0.923
Diabetes mellitus, *n* (%)	2 (20%)	1 (5.3%)	0.267
Pulmonary disease, *n* (%)	0	1 (5.3%)	1.000
Onset to hospital admission, days, median (IQR)	6 (3–17.5)	4 (3–7)	0.178
Hospital admission before day 14, *n* (%)	7 (70%)	18 (94.7%)	0.105
GDS at admission, median (IQR)	3 (2.75–4)	4 (2–4)	0.701
GDS > 2 at admission, *n* (%)	8 (80%)	12 (63.2%)	0.431
MRC-SS at admission, median (IQR)	41.5 (38–44.5)	46 (39–54)	0.174
Cranial nerves involvement at admission, *n* (%)	2 (20%)	6 (31.6%)	0.675
Facial weakness at admission, *n* (%)	2 (20%)	7 (36.8%)	0.431
Bulbar weakness at admission, *n* (%)	2 (20%)	6 (31.6%)	0.675
Sensitive disturbances at admission, *n* (%)	10 (100%)	13 (68.4%)	0.068
Limb weakness at admission, *n* (%)	10 (100%)	18 (94.7%)	1.000
Autonomic dysfunction during hospitalization, *n* (%)	3 (30%)	4 (21.1%)	0.665
Onset to nadir, median (IQR), days	10 (5–14)	8(4–10)	0.054
Nadir before day 14, *n* (%)	7 (70%)	17 (94.7%)	0.116
GDS at nadir, median (IQR)	3.5 (2.75–4.25)	4 (2–5)	0.670
GDS > 2 at nadir, *n* (%)	8 (80%)	14 (73.7%)	1.000
Onset to lumbar puncture, median (IQR), days	17 (11–27)	13 (12–15)	0.154
CSF proteins, median (IQR), mg/dL	1.07 (0.83–3.32)	0.64 (0.46–1.19)	0.035
CSF proteins > 0.5 mg/dL, *n* (%)	8/9 (88.9%)	11/14 (78.6%)	1.000
WBC in CSF, median (IQR), *n*/µL	2 (0–3)	2 (0.75–10.25)	0.235
WBC in CSF > 10/µL, *n* (%)	0/9	3/14 (21.4%)	0.253
Onset to NCS, median (IQR), days	26 (13–40)	18 (14–22.25)	0.090
NCS findings			
AIDP, *n* (%)	7 (70%)	14 (73.7%)	0.486
AMAN, *n* (%)	1 (10%)	2 (10.5%)	
AMSAN, *n* (%)	0	2 (10.5%)	
Non-defined, *n* (%)	2 (20%)	1 (5.3%)	
Therapy			
IVIg, *n* (%)	10 (100%)	16 (84.2%)	
PLEX, *n* (%)	0	1 (5.3%)	0.415
Symptomatic, *n* (%)	0	2 (6.9%)	
Mechanical ventilation, *n* (%)	2 (20%)	4 (21.1%)	1.000
Duration of mechanical ventilation, median (IQR), days	9.5 (8–11)	47.5 (29.5–79.5)	0.064
GDS on discharge, median (IQR) (survived)	3.5 (1.75–4)	3 (1–4)	0.565
Adverse outcome, *n* (%)	0	3 (15.8%)	0.532
Hospital length of stay, days, median (IQR) (survived)	16 (9–25)	18 (12–27)	0.33
Place of discharge			
Home, *n* (%)	4 (40%)	6 (37.5%)	
Rehabilitation facility, *n* (%)	3 (30%)	3 (18.8%)	0.723
Other hospital, *n* (%)	3 (30%)	7 (43.8%)	

*GBS* Guillain–Barré syndrome, *GDS* Guillain–Barré syndrome disability score, *MRC-SS* Medical Research Council sum score, *CSF* cerebrospinal fluid, *WBC* white blood cell, *AIDP* acute inflammatory demyelinating polyneuropathy, *AMAN* acute motor axonal neuropathy, *AMSAN* acute motor–sensory axonal neuropathy, *IVIG* intravenous immunoglobulin, *PLEX* plasma exchange

In our study there was no statistically significant difference in the time from the symptom onset to lumbar puncture (17 days in post-COVID-19 patients vs. 13 days in non-COVID-19 patients) (*p* = 0.18). Although there was no difference in the percentage of patients with CSF protein level > 0.5 mg/dL between these two groups (*p* = 1.00), higher protein levels in CSF were observed among post-COVID-19 GBS patients (1.07 mg/dL vs. 0.64 mg/dL, *p* = 0.035). NCS showed no difference in the subtype of GBS between these two groups (*p* = 0.486). The most frequent subtype of GBS in both groups was AIDP.

The therapeutic approach also did not differ between these two groups of patients (*p* = 0.42). Two (20%) of post-COVID-19 GBS and four (21.1%) of non-COVID-19 GBS patients required MV during hospitalization (*p* = 1.00). Duration of MV did not differ between these two groups (*p* = 0.064). Symptoms of autonomic dysfunction (oscillation of blood pressure and tachycardia) were noted in 3 (30%) patients in post-COVID-19 group and 4 (21.2%) patients with non-COVID-19 GBS. In both groups symptoms of autonomic dysfunction resolved during hospitalization. All of our post-COVID-19 GBS patients survived, whereas three (15.8%) of non-COVID-19 GBS patients had lethal outcome (*p* = 0.532). In three patients with non-COVID-19 GBS, lethal outcome was caused by multiorgan failure caused by complications of ICU hospitalization such as infection.

GDS at discharge (*p* = 0.56), as well as length of hospitalization (*p* = 0.33), did not differ between these two groups. There was no difference in GDS at 3 months of follow-up 3 months after discharge between non-COVID-19 and post-COVID-19 patients (*p* = 0.22).

## Discussion

Our study was conducted in order to provide additional information on the clinical course and outcome of GBS following COVID-19 infection. Over a one-year period, we detected 10 patients diagnosed with GBS triggered by COVID-19 and 19 patients with GBS due to other or unknown preceding events. Considering the fact that our hospital is not a COVID-19 treating medical institution, none of our patients had an active COVID-19 infection at the time of admission. Therefore, our study refers to post-infective GBS. However, cases of GBS during an active COVID-19 infection were also reported in the literature [20, 21]. Our study was conducted from March 2020 to March 2021, and vaccination against COVID-19 in our country for general population started in January 2021, that is why only two patients who were admitted in January and February were vaccinated, and both were administered one dose of Sinopharm Vero Cell vaccine.

Most of our patients were older than 50 years which is in accordance with previous observations referring to both GBS in the general population and post-COVID-19 GBS [22]. Furthermore, older patients were shown to be more prone to SARS-CoV-2 infection and GBS has its peak incidence in the population between 50 and 69 year of age [23, 24].

The median time from the preceding event to the onset of neurological symptoms was longer in the cohort of post-COVID-19 compared to non-COVID-19 GBS patients. This could be the result of the selection bias based on the fact that all our patients required negative PCR of nasopharyngeal swab for SARS-CoV2 at the admission and it has been shown that PCR can remain positive for one month after exposure to SARS-CoV-2 [25].

Our study revealed no difference in the clinical features of the studied groups. These results are in accordance with recent studies conducted in Great Britain and Italy, which concluded that the pattern of weakness and sensory disturbances were not different in COVID-19 GBS patients compared to non-COVID-19 GBS patients [11, 12].

The most frequent subtype of GBS in both our groups was AIDP. Unrelated to the preceding event, AIDP is the most common subtype of GBS present in about 69–90% of patients [22] and it is also the most common subtype among GBS patients in our country [26]. This clinical variant has also been reported as the most frequent subtype of GBS associated with COVID-19 [12, 27, 28]. There was no difference in the representations of GBS subtypes between post-COVID-19 and non-COVID-19 GBS patients which is in line with the results of the study conducted in Great Britain [12]. However, one Italian study reported significantly higher prevalence of AIDP in patients with COVID-19-associated GBS compared with non-COVID-19 GBS patients [11]. These results might be a consequence of the small number of non-COVID-19 GBS patients in this study [11].

Regarding CSF findings, there was no difference in the white blood cell count, but higher protein level in CSF was noted in post-COVID-19 GBS patients. Higher concentration of protein in the CSF has been associated with increased permeability of blood–brain barrier [29], therefore, we presume that higher proteinorrachia might occur due to direct neuroinvasive abilities of the SARS-CoV2 virus or neuroinflammation [30, 31]. A recently published study, which analysed the findings of CSF in patients with neurological manifestations of COVID-19, reported elevated cerebrospinal fluid protein levels in three-quarters of the patients, almost half of whom were diagnosed with GBS [32]. The theory of higher proteinorrachia due to a disrupted blood–brain barrier is also supported by the results of a recent study that showed the presence of anti-SARS-CoV2 antibodies in the CSF of 8 patients with the signs of encephalopathy during COVID-19 infection [33].

Disease severity and outcome did not differ between studied groups. Furthermore, we did not notice a difference in the need for MV and its duration, which is in contrast to the results of recent studies that showed that patients with GBS associated with COVID-19 are more likely to require MV [32]. We did not include patients with active lung disease, while other authors did, which could explain such a difference [12, 34].

There was no difference in chosen treatment modality among studied groups. All post-COVID-19 and 16 (84.2%) of non-COVID-19 patients were treated with IVIG. Among the remaining three non-COVID-19 GBS patients, one underwent plasma exchange, one had contraindications for specific treatment due to a recent myocardial infarction, and one had a mild clinical presentation with a good response to symptomatic therapy.

Results of our research imply that GBS developed after COVID-19 should be treated and monitored in the same manner as GBS caused by other preceding factors because we observed no difference in clinical and electrophysiological features and outcomes between studied groups. Presence of greater proteinorrachia in post-COVID GBS might be clinically relevant, particularly in context of borderline longer onset to nadir time in the same group. It might reflect COVID-19 infection impact as a dominant pathophysiological pathway compared to other post-infective non-COVID-19 GBS forms.

The main limitations of our study are a small number of patients and selection bias due to the fact that patients with active COVID-19 infection were not included. Furthermore, to obtain more objective results, it would be necessary to conduct a multicentric study that would include COVID-19 treating institutions. However, if patients with active COVID-19 infection and parainfectious GBS were included, the combined effect of both diseases on patient outcomes should be assessed.

## Conclusions

There was no difference in clinical and electrophysiological features, disease course, and outcome between post-COVID-19 and non-COVID-19 GBS patients.

## Data Availability

The data that support the findings of this study are available on request from the corresponding author. The data are not publicly available due to privacy or ethical restrictions.

## Abbreviations

GBS: Guillain–Barré syndrome
MRC-SS: Medical Research Council sum score
GDS: Guillain–Barré syndrome disability score
MV: Mechanical ventilation
NCS: Nerve conduction studies
CSF: Cerebrospinal fluid
AIDP: Acute inflammatory demyelinating polyneuropathy
AMAN: Acute motor axonal neuropathy
AMSAN: Acute motor-sensory axonal neuropathy
IVIG: Intravenous immunoglobulin
PLEX: Plasma exchange
